# Anticipating the future of the child and family in pediatric palliative care: a qualitative study into the perspectives of parents and healthcare professionals

**DOI:** 10.1007/s00431-020-03824-z

**Published:** 2020-10-08

**Authors:** Lisa M. Verberne, Jurrianne C. Fahner, Stephanie F. V. Sondaal, Antoinette Y. N. Schouten–van Meeteren, Chris C. de Kruiff, Johannes J. M. van Delden, Marijke C. Kars

**Affiliations:** 1 Department of pediatrics, Catharina Hospital, Michelangelolaan 2, 5623 EJ Eindhoven, The Netherlands; 2 Julius Center for Health Sciences and Primary Care, University Medical Center Utrecht, Heidelberglaan 100, 3508 GA Utrecht, The Netherlands; 3 Princess Máxima Center for Pediatric Oncology, Lundlaan 6, 3584 AE Utrecht, The Netherlands; 4 Emma Children’s Hospital, Amsterdam University Medical Centre, Meibergdreef 9, 1105 AZ Amsterdam, The Netherlands; 5 Center of Expertise Palliative Care, Julius Center for Health Sciences and Primary Care, University Medical Center Utrecht, Utrecht University, Heidelberglaan 100, 3508 GA Utrecht, The Netherlands

**Keywords:** Communication, Advance care planning, Goals of care, Pediatric palliative care

## Abstract

Preparing for future scenarios in pediatric palliative care is perceived as complex and challenging by both families and healthcare professionals. This interpretative qualitative study using thematic analysis aims to explore how parents and healthcare professionals anticipate the future of the child and family in pediatric palliative care. Single and repeated interviews were undertaken with 42 parents and 35 healthcare professionals of 24 children, receiving palliative care. Anticipating the future was seen in three forms: goal-directed conversations, anticipated care, and guidance on the job. Goal-directed conversations were initiated by either parents or healthcare professionals to ensure others could align with their perspective regarding the future. Anticipated care meant healthcare professionals or parents organized practical care arrangements for future scenarios with or without informing each other. Guidance on the job was a form of short-term anticipation, whereby healthcare professionals guide parents ad hoc through difficult situations.

*Conclusion*: Anticipating the future of the child and family is mainly focused on achievement of individual care goals of both families and healthcare professionals, practical arrangements in advance, and short-term anticipation when a child deteriorates. A more open approach early in disease trajectories exploring perspectives on the future could allow parents to anticipate more gradually and to integrate their preferences into the care of their child.

**Table:** 

**What is Known:** *• Anticipating the future in pediatric palliative care occurs infrequently and too late.*	
**What is New:** *• Healthcare professionals and parents use different strategies to anticipate the future of children receiving palliative care, both intentionally and unwittingly. Strategies to anticipate the future are goal-directed conversations, anticipated care, and guidance on the job.* *• Parents and healthcare professionals are engaged to a limited extent in ongoing explorative conversations that support shared decision-making regarding future care and treatment.*

**Electronic supplementary material:**

The online version of this article (10.1007/s00431-020-03824-z) contains supplementary material, which is available to authorized users.

## Introduction

The number of children with life-limiting or life-threatening conditions (Box 1) is increasing as current medical treatment options allow critically ill children to live longer, being dependent on high-complex care for a longer period of time and expanding care facilities at home [1–4]. These children are in need of pediatric palliative care (PPC) from the point of diagnosis and continued throughout the child’s life and death [1, 5]. During different disease trajectories, preparing for future scenarios is perceived as complex and challenging by both families and healthcare professionals (HCPs) [6–9]. For parents, facing the future is emotionally challenging as it confronts them with the possible loss of their child [9, 10]. By discussing the future with families, HCPs fear to take away hope and disturb the families’ way of coping with the serious illness of their child [7, 8]. These factors may result in refraining from facing the future leading to a delayed initiation of PPC and insufficient attention to the child’s quality of life (QoL), especially at the end-of-life [11]. However, growing evidence shows that both families and HCPs value strategies to explore future scenarios in advance [10, 12–15]. In recent literature, there is growing interest in the concept of advance care planning (ACP) as a strategy to identify goals and preferences for future care and treatment, to share these thoughts between families and HCPs and document any preferences if considered appropriate [16]. Yet, it is known that ACP in pediatrics occurs infrequently and often too late due to barriers on the level of families, HCPs, and healthcare organizations [6–8, 13]. Limited research is done on current strategies of facing the future as used by HCPs and families when caring for a seriously ill child. We hypothesize that different ways of anticipating the future of the child and family may occur. Insight in current approaches of anticipating the future in PPC is needed. Based on these insights, strategies can be further developed to elicit individual family’s values and preferences for future care and treatment in order to support high quality family-centered care from diagnosis of a life-limiting condition until the end-of-life. Therefore, this study aims to explore how parents and HCPs currently anticipate the future of the child and family in PPC.

Box 1 Definitions [1]

**Table:** 

Life-limiting disease: conditions for which are that there is no reasonable hope of a cure and from which children or young people will die. Life-threatening disease: conditions for which are that curative treatment can be feasible but can fail.	

## Methods

### Study design

As part of a larger study exploring the lived experience of families receiving PPC and their HCPs involved, an explorative qualitative study was conducted using inductive thematic analysis to elucidate approaches of anticipating the future of the child and family among parents and HCPs. The research ethics committee of the Academic Medical Centre Amsterdam approved the study (2013; Reference number: W13_120#13.17.0153). All participants gave written informed consent.

### Sample

Parents of children with a life-limiting condition, receiving care from the pediatric palliative care team (PPCT) of the Emma Children’s Hospital, were purposefully selected. Maximum variation was sought with respect to the child’s diagnosis, age, and disease trajectory, including end-of-life [17–19]. Parents could also be included after the child’s death to achieve insight in very last period of life. PPCT case managers as well as other HCPs most involved in each selected case were also recruited.

### Data collection

Parents were individually interviewed at home and HCPs at their workplace or by telephone between August 2013 and January 2016. Interviews lasted from 30 min to 2 h. They were conducted by independent researchers (LV, MK, MB) from a university hospital other than where the PPCT was established. A topic list based on literature and expert knowledge guided each interview (Supplementary information; Topic list 1 and 2). The interviewer explored how and to what extend parents and HCPs anticipated the future of the child and family in PPC and how they experienced this. Audio recordings of the interviews were anonymously transcribed verbatim.

### Data analysis

The data was analyzed using inductive thematic analysis [20–22]. Validity was ensured by a rigorous study design and repetitive meetings of the research team (LV, JF, SS, and MK). An audit trail recording methodological choices and substantive ideas and concepts related to the interpretation of the data was used to further ensure validity and provide transparency of the results.

The analysis yielded four steps. First, transcripts of five cases were (re)read to gain an overall understanding of the study objectives in context of the interviews. Meaningful fragments were identified in all five interviews. These fragments were coded in a data-driven manner (LV, SS, and MK) [20]. Second, of each interview, a narrative report was made to summarize strategies to approach future care. Fragments, initial codes, and summaries were compared and discussed aimed at reaching consensus in interpretation. The initial codes were combined, recoded, and adapted towards a code tree with themes and concepts at a more abstract and conceptual level. Third, all interviews were coded using NVivo10 [23]. After coding each case, the coding tree was evaluated and, if indicated, revisited. Fourth, based on the code tree, potential themes were identified. These were consistently verified, reviewed, and refined on coherency by constant comparison of the data per theme and of the whole thematic map in relation to all the data [21]. Saturation was reached at a conceptual level [24]. The Consolidated Criteria for Reporting Qualitative Research was used to structure the report [25].

## Results

Of the 35 cases eligible for participation, 24 were included, resulting in the participation of 42 parents (24 mothers and 18 fathers) and 35 HCPs. Reasons for non-participation were parental refusal (*n* = 5) and HCPs considering a case too vulnerable to participate (*n* = 6). Three cases were included after the child’s death (parents, *n* = 6; HCPs, *n* = 10) and in three other cases, a repeated interview with the parents (*n* = 5) and with HCPs (*n* = 7) was done after the child’s death. Several HCPs were involved in multiple cases and, thus, interviewed several times. In total, 105 semi-structured interviews were conducted (parents, *n* = 47; HCPs, *n* = 58). For participant characteristics, see Tables 1 and 2.

**Table 1:** Characteristics of the parents (*n* = 42) and their ill child (*n* = 24)

Characteristics	Number (*n*)	Percentages (%)
Gender parent		
Male	18	43
Female	24	57
Age parent^a^		
< 30	2	5
30–40	29	73
> 40	9	23
Marital stage		
Married/cohabiting	38	90
Divorced/not cohabiting	4	10
Education		
Low^b^	5	12
Middle^c^	15	36
High^d^	22	52
Age child (at first interview) (years)		
0–1	1^e^	4^e^
1–5	13^f^	54^f^
5–12	7	29
12–16	2	8
≥ 16	1	4
Child gender		
Male	12	50
Female	12	50
Child diagnosis		
Non-malignant disease (total)	15	63
Congenital anomalies	11	46
Neurodegenerative disease	2	8
Metabolic disease	2	8
Malignant disease (total)	9	38
Central nervous system tumor	5	21
Bone/soft tissue sarcoma	2	8
Neuroblastoma	1	4
Leukemia	1	4
Time since diagnosis		
0–6 months	2	8
6–12 months	3	13
1–2 years	7	29
2–5 years	8	33
> 5 years	4	17
Palliative phase at first interview		
Diagnostic phase	0	0
Phase of loss of normality	15	63
Phase of decline	6	25
Dying phase	3	13

Percentages may not equal 100 due to rounding

^a^Age of two parents is missing

^b^Low: primary school, lower secondary general education, lower vocational education

^c^Middle: higher secondary general education, intermediate vocational education

^d^High: higher vocational education, university

^e^In one case, the interview took place after the child’s death

^f^In two cases, the interview took place after the child’s death

**Table 2:** Characteristics of the healthcare professionals (HCP) (*n* = 35)

Characteristics	Number (*n*)	Percentages (%)
Vocation HCP		
Pediatrician^a^	14	40
Pediatric revalidation specialist	1	3
General practitioner	5	14
Case managers (PPCT nurse)	6	17
Homecare nurse	7	20
Other^b^	2	6
Years of working experience in palliative care?		
0–5 years	5	14
6–15 years	9	26
> 15 years	19	54
Unknown	2	6

^a^General pediatrician (6), pediatric oncologist (5), pediatric neurologist (2), pediatric intensivist (1)

^b^Psychologist of the PPCT (1) and child-life specialist of the PPCT (1)

### Anticipating the future

Many parents and HCPs experienced anticipation of the future of the child and family as difficult because of uncertainties due to the unpredictability of the disease course. Moreover, it required acknowledgement of disease progression and facing the child’s inevitable death. Despite these difficulties, parents as well as HCPs were seen to anticipate future care and treatment. Initiatives to share perspective were predominantly aimed at ensuring the child’s quality of life and comfort, also during the end-of-life. However, individual perspectives regarding the future were not shared between parents and HCPs to a large extent. Three forms of future anticipation were revealed: goal-directed conversations (GDC), anticipated care (AC), and guidance on the job (GOTJ). For illustrating quotes, see Table 3.

**Table 3:** Illustrative quotes of goal-directed conversations, anticipated care, and guidance on the job taken from the interviews

Aspects of goal-directed conversations	
Goals	
Parents	MD, case 23, father: *For me, he (son) does not have to suffer pain. In the end that is not what we want. […] We have indicated to the oncologist that in case something happens and he has to be resuscitated, that we do not want that because he will not survive it well. […] We really chose quality of life.*
HCPs	MD, case 20, pediatrician: *I discussed in the beginning, whether she would go to an ICU, [have] a DNR order. Parents were both very clear about it, which made it easier for me, no resuscitation and aiming for comfort.*
Parents’ varying strategies to cope with anticipated loss	
Parents	NMD, case 7, mother: *Now that he is in such an advanced stage of the disease, and possibly because of my own character, I need to know [what I can expect in the future]. I do not live in the future, but I need to know, I need to understand. I somehow need to prepare myself, because for me it is also important to touch [the future] and see how that feels, because I have the feeling that if I do not do this, I will not survive the blow that is coming.*
HCPs	MD, case 13, pediatrician: *The father’s character is one of ‘what if, what if’. And the mother is much more the one who says ‘yes, yes’, and who gives me the feeling that she sometimes would rather not talk about it. They are two different people in this respect. It happens that father addresses me separately, he does a literature search […] and refers back to parts of the talks we have had before.*
Framing	
HCPs	MD, case 5, pediatric oncologist (after marking end of curative phase): *What I usually try to do is a sort of looking ahead. The emphasis will often lie on the first weeks, but […] we always [try] to make a sketch of later phases. […] And later we go into those more deeply, when they are ready for it, but it is good to know that that phase will come, that we sometimes already have to take measures for that now. But talking about this also helps, […] to already prepare them for it. The next time we meet, I’ve noticed parents come back with a lot of questions. And in such a way you color in the drawings more and more, the closer it gets.*
Revisiting discussions on future treatment	
Parents	NMD, case 8, mother: *I feel that Pim [son] is doing better than [the doctors] ever expected. So, then I believe it [decisions] should be adjusted, not regarding not resuscitating, […] if the heart would stop, it stops and then you might create more damage [if you would resuscitate]. But for example, with intense pneumonia, and you think he just needs help a little longer, then I would like him to be given supportive respiration.*
Anticipated care	
Closed	
Parents	MD, case 5, father: *The conversation with the lady working at the funeral company, I initiated it myself because I found it important to start with that on time. So, I looked for contacts in the neighborhood and it [meeting] was organized in a flash. And she [funeral organizer] found it very valuable, despite that it was a very unclear trajectory, […because] they could think ahead already now, or Pieter [son] can indicate for himself what he likes.*
HCPs	NMD, case 18, PPCT nurse: *At some point, he [child] will be able to do so little that he will give up. […] And I think that when certain things are no longer possible at some point, he will quit. I hope that that will still take some time, but it is not for him to get into a vegetative state […] My goal with him is, maybe a bit weird, [but] prepare him for death. I would want and [organize] someone [to] get into contact with him about the nearing end and the process of losing all that he could do.*
Open	
Parents	NMD, case 21, mother: *During the last admission, […] I said then [that] I just do not dare take her home before I learn how to do deeper suction and how to resuscitate. Because when something happens to her [daughter], I want to be able to do something. […] That was a difficult topic, because the pediatrician was thinking […] how am I sending a parent home, with so many worries. But what is sometimes not understood is that you would send a parent home with even more worries when they are not able to resuscitate.*
HCPs	NMD, case 12, pediatrician: *Then we thought with the PPCT, what if he has pain, what if he becomes dyspneic, what if he gets a seizure, how will we treat that medically, who will we involve with the care for this patient. […] Then we wrote a palliative protocol together and […] visited the two family doctors […and] made agreements on who would do what. […] And only when you have that clear, you discuss those steps with parents.*
Guidance on the job	
Parents	MD, case 22, mother (about the further deterioration of her child): *I find it comforting that those thoughts occur in steps and that the emotions also surface in steps. You are being taken by the hand [by the specialized nurse of the PPCT] a bit to look at the situation more from a meta level and to think about and make decisions together, for things that will come but not just yet. […] I think that that is good because […] now you can do it in a well thought-out manner.*
HCPs	MD, case 5, homecare nurse (when child becomes increasingly dyspneic): *He [child] of course did not want anything, he preferred to wait [what would come]. Then I discussed, ‘you [child] are now so uncomfortable, this is not pleasant’. And the parents also said, this is also not what we want. […] We have discussed it, there are many possibilities to make you [child] calmer. So, I am very open and discuss why I want to do it [start with morphine]. But I have also said that he will not die from the morphine plaster. […] Then we gave him extra medication because he [child] was very uncomfortable and told them that we would start the pump tomorrow and possibly tonight if things do not improve.*

Some quotes are slightly modified to improve readability. Names are fictitious

*DNR* do not resuscitate; *HCPs* healthcare professionals; *ICU* intensive care unit; *MD* malignant disease; *NMD* non-malignant disease; *PPCT* pediatric palliative care team

### Goal-directed conversations

Initial conversations, both initiated by HCPs or parents, on future care and treatment as a way of sharing each other’s perspectives appeared not to occur naturally. Rather, these conversations regarding future scenarios had a conscious and goal-directed intention. In order to align the perspective on future care and treatment, both HCPs and parents shared their views on care and treatment in the future to the other party in the conversation. Initiation of such a conversation and mutual alignment of these care goals proved essential to influence the other party’s willingness to adapt their perspective and actions.

#### HCPs

Usually HCPs took the initiative to start a conversation regarding future care or treatment. HCPs mentioned to initiate a conversation about future care and treatment driven by ethical reasons, such as to prevent medically futile treatments or to ask consent for advance directives. They mentioned practical conversation goals as well, such as to have clarity about the preferred place of death. Although HCPs mentioned to explore the parents’ perspective in the conversation, they reported to have clear ideas about future care and treatment in advance. These care goals from the HCP’s perspective were mostly based on their own perspectives or on discussions within the medical team.

Besides their aim for getting parental consent on future care and treatment options, HCPs mentioned talking about the future were also aimed at preparing parents for difficult decision-making to be expected in the future. Some HCPs mentioned to initiate a conversation about the future, when they felt the parent had an unrealistic and too positive view on their child’s condition.

In order to create a shared perspective on the child’s condition, HCPs marked new stages of the disease trajectory in goal-directed conversations by emphasizing a concrete or objective aspect in the child’s condition or disease trajectory that clearly indicates that the child had entered or will enter in the future a new stage in the disease trajectory. This required from parents to reconsider their views on future care. HCPs either marked actual situations in the moment or prepared parents to expect marking moments in the future. Examples were the failure of cancer treatment indicating a shift from disease-directed treatment towards symptom-directed treatment or a hospital admission due to deterioration of the child, indicating the child’s increased vulnerability. Based on these marked new stages of the disease trajectory, HCPs framed parents by discussing the child’s condition in relation to these different stages and possible options for care and treatment, in order to clarify consequences for the child, for example, framing the high likelihood of a pediatric intensive care unit admission when continuing treatment or the negative consequences of resuscitating children given their condition.

#### Parents

Parents took the initiative to start a conversation about the future of their child in order to achieve a good life for their child with the least amount of suffering as possible. Another reason to discuss their future with HCPs could be parental goals of continuing regular family life and to receive clues around the prognosis of their child based on the HCPs’ expertise. Parents needed the knowledge and insights of the HCP to be able to arrange the care for their child for a longer period of time and to be able to develop their perspectives on family planning. Parents also needed the HCP’s formal approval to get access to care arrangements, such as modifications to their homes. The abovementioned goals were mainly reported by parents with a focus on prolonging the child’s life as well as by parents with a perceived longer life expectancy of their child.

Those parents who had a focus on comfort care without striving for prolonging life initiated conversations about their child’s future to be able to cope with their own ongoing loss. Some parents reported to start a conversation about future care in order to prevent their child’s suffering and unnecessary prolongation of life. These parents sought HCPs’ expertise, guidance, and agreement on limitation of life-sustaining treatments and options to allow a natural death. Parents whose HCP had been easily approachable felt more openness to ask questions about delicate issues, such as when to stop tube feeding and what could occur during the dying phase of the child. Some parents reported that HCPs had not been open for exploring the future or answering their questions, mainly by referring to prognostic uncertainty.

Few parents reported to initiate a conversation about the future aimed at reconsidering prior treatment limitations written down in an advance directive. These parents had observed a clear, yet unexpected improvement in the child’s condition, which in their opinion justified revisiting treatment limitations. Some parents framed the new situation towards HCPs as they felt a need to place the child’s condition in relation to a broader contact of disease course and treatment options. As such, they tried to convince HCPs to align to their perspective and goal setting as a parent with expertise on their child’s condition. Parents felt a need to place the child’s condition in relation to a broader context of disease course and treatment options in order to convince HCPs to align to their perspective and goal setting as a parent with expertise on their child’s condition.

Overall, the parents’ way of coping with the future loss of their child influenced their ability to discuss future care and treatment. Parents, who tend to focus on the “here-and-now” to be able to cope with feelings of loss and the daily burden of care, experienced difficulties or refused to discuss future care and treatment with HCPs.

### Anticipated care

AC involved being prepared for future scenarios by shaping and organizing care arrangements in advance, in response to anticipated future needs of the child or family. AC occurred during the different disease stages in a similar way, although the content of AC might vary according to the focus of care, the changes in the child’s condition, and the preferences of the child and family. AC was mostly initiated by HCPs and sometimes by parents. It had either a “closed” or “open” character depending on whether HCPs or parents informed each other about the care arrangements made. Disclosure of “closed” AC occurred when a need arose among either HCPs or parents to inform each other about the preparations.

#### HCPs

AC was mainly conducted by HCPs experienced in PPC, such as PPCT members or pediatric homecare nurses, and often discussed among HCPs preparing for future care without informing the parents at the time. Examples included ordering medications and equipment for the home setting, creating a contact plan for parents, and involving other important HCPs, such as the PPCT, general practitioner, psychologist, or child-life specialist. HCPs often started with “closed” AC, mainly to prevent unnecessary burden to the parents or to prevent disruption of the parental coping strategy. Disclosure of “closed” AC occurred when parents were perceived as ready for the intended care arrangements or when the HCPs perceived the child’s or the parents’ interest as threatened when withholding the planned care. The tuning and timing when to provide insight in “closed” AC arrangements were experienced as a delicate task, preventing that care would be provided too late or started too early.

#### Parents

Only a few parents seemed to prepare for the future by organizing care arrangements in advance. Parents also used “closed” or “open” AC. Parents only informed HCPs when HCPs invited them to do so or when parents needed help from HCPs to arrange the care they aimed for. An example of “closed” AC performed by parents is organizing their child’s funeral in advance without mentioning this to their HCP. An illustration of “open” AC was a mother requesting a resuscitation course from the pediatrician to become able to take care of her daughter at home during an emergency.

### Guidance on the job

GOTJ was discerned as a form of short-term anticipation on scenarios or symptoms to be expected in the near future. This form of anticipating the future was only conducted by HCPs. HCPs guided parents by explicitly informing them about the child’s current situation and short-term expectations thereof, indicating the necessity why certain actions or approaches were required now or in the near future. In this way, HCPs aimed to prepare parents what to expect among the deterioration of their child and needed care and how to act in the expected situation.

Most examples of GOTJ were related to moments of acute deterioration of the child or situations where death was imminent. HCPs used GOTJ to help parents to provide care aligned to the child’s altered needs. It was done in situations where parents seemed to be at risk to overlook new care needs of the child or felt unable to adequately respond to them. This could be a result either of inexperience or of difficulties in coping with the child’s end-of-life. This included for example being afraid to hasten the child’s death by starting morphine of withdrawal of feeding. GOTJ was both child-focused, aimed at improving the child’s comfort, as well as parent-focused, aimed at coaching and supporting parents to “be there” for their child and to act in the best interest of their child in situations that were difficult to predict or hardly bearable.

Parents indicated appreciation of GOTJ. It made them feel supported and helped them to cope with uncertain future scenarios. It prepared and enabled them to go through difficult steps in the disease trajectory of their child. Some parents felt relieved that HCPs took the lead to proceed in the end-of-life process, not wanting the final responsibility for decisions regarding the child’s end-of-life, such as treatment limitations, start of palliative sedation, or end of feeding.

## Discussion

Parents and HCPs faced the future of the child and family to a various extent when caring for a child receiving palliative care. Parents and HCPs anticipated the future in order to safeguard the child’s quality of life, comfort, and quality of death, and to maintain family balance. Three forms of anticipating the future were identified: goal-directed conversations, anticipated care, and guidance on the job. The parents’ coping with the anticipated loss of their child’s abilities and, ultimately, their child’s life and the expertise of HCPs to support parents in facing the future largely influenced the occurrence of GDC and the need for AC and GOTJ.

In current research and practice in the field of PPC, there is a growing interest in strategies to anticipate the future in an adequate way [26]. Tools are developed to support families and HCPs in anticipating the future by ACP [27, 28]. Our study reveals some key points that show why implementation of ACP and other strategies to anticipate the future need ongoing attention.

A central element of newly developed ACP strategies is the identification of individual values and preferences that can inform shared decision-making about goals of future care and treatment. In the cases in the current study, anticipating the future in an open and explorative way aimed at the identification of patient values occurred to a limited extent. This study also showed that currently parents and HCPs shared future perspectives mainly when they considered it necessary to safeguard care goals or because the involvement of the other was indispensable. Consequently, these conversations of sharing future perspectives rather had a directional, than an open, explorative character. Given the current family-centered ideal of providing PPC [1, 5], anticipating the future might need strategies to explore families’ values and preferences for future care, in addition to a goal-directed approach as conducted currently by HCPs as observed in our study [13, 16]. An open and explorative approach could facilitate shared decision-making and allow for an earlier and more gradual integration of families’ values and preferences in future care and treatment as is aimed for in ACP. It is known that parents value ACP, yet they might hesitate to share their values and preferences for their child’s care and treatment by themselves [9, 29, 30]. Families often wait for the HCP to start conversations about future care [31, 32]. As such, it might be helpful when ACP tools support HCPs to invite parents for ACP conversations and help them to achieve an open and explorative approach when anticipating the future in conversations.

It is known that parents experience anticipating the future as an inevitable part of seeking good care for their child, although it confronts them with ongoing losses [9, 10, 33]. Our study showed that all parents, even parents who coped with distress by living day-by-day in the present, regularly had thoughts about their child’s anticipated early death. This knowledge should stimulate HCPs to explore these perspectives and open up a conversation about what is important to families facing the child’s possible death. Also uncertainty due to the unpredictable course of the disease [6, 8, 9, 18], which is known as a barrier to anticipate the future for both parents and HCPs, should rather be a trigger for conversations about future care and treatment in order to start these conversations in time, as also previously argued by Kimbell et al. [29]. Timely initiation of these conversations would allow parents a well-timed transition from an attitude of preserving their child at all costs towards letting go when time has come [30].

From our study, we could identify GDC, AC, and GOTJ as three separate forms to anticipate future care and treatment. In practice, these three forms may intermingle, occur simultaneously, and ideally merge into each other. All strategies can be part of an ACP process, in which different phases in the child’s disease trajectory lead to different coping mechanisms requiring a specific approach. For example, during the end of life of the child or an acute deterioration of the child’s condition, families might need more guidance from HCPs, requiring GOTJ. In cases with little or no GDC or AC, occurrence of GOTJ was more prominent and required HCPs to keep track of the child’s situation more actively to identify any changes in time. If GOTJ was not performed actively, adequate childcare could be addressed too late, again emphasizing the importance of timely initiation of ACP [29, 30]. Ideally, GOTJ can consecutively build on earlier GDC and benefit from well-organized AC. AC that was based on the HCPs’ or parents’ own perspective mainly could be better aligned to the child’s and family’s needs and wishes when prior conversations had elicited their values. As such, GDC, AC, and GOTJ ideally co-exist and will rely on prior discussions and to the child’s and family’s actual needs. Moreover, well-performed anticipation of the future of the child and family by both parents and HCPs offers an important fundament for shared decision-making [12].

Kimbell et al. and other studies [8, 29] also highlighted the importance of a continuing process in ACP, with regular reviewing preferences and goals of care. In this study, parents initiated revisions of previously made agreements, such as advance directives, when they saw their child’s condition improved. HCPs regularly discussed the child’s current state with parents but whether they monitored changes in parents’ perspectives on future care and treatment was less clear. Research for future ACP interventions can investigate how to incorporate regular monitoring and, if needed, revisions of preferences for care and treatment.

This study had some strengths and limitations. Being a one-centered study, the generalizability of our results might be limited. Nevertheless, purposeful sampling facilitated a wide variation regarding diagnosis, age, and phase of palliative trajectory. This research also offers a broad and diverse perspective on data from 24 cases crossing different age groups and including insights of both parents and HCPs. Some HCPs regarded few eligible parents to be too burdened to participate, preventing or delaying their inclusion. This is known as gatekeeping and often seen in palliative care research [34]. This aspect might have resulted in an overestimation of the occurrence of GDC, AC, and GOTJ and an underestimation of parents who have difficulties to anticipate future care. We did not capture differences in cultural and religious aspects, which is a limitation because there are cultural differences in decision-making and communication styles [35]. Our findings might be limited by not analyzing recordings of the actual conversations between parents and HCPs; however, the interviews were believed to give valuable insights into perspectives regarding anticipation of the future. Future research could focus on the implementation of ACP to anticipate the future of the child and family in a more comprehensive way, while exploring values and preferences for the future without any need for achieving goals, decision-making, or arranging care at that moment. Perspectives shared in ACP can function as a foundation for the content of GDC, AC, and GOTJ, which might remain necessary in certain situations, even when adequate ACP occurred in advance.

## Conclusion

This study showed that parents and HCPs anticipate the future of the child and family in PPC mainly by GDC, AC, and GOTJ. Sharing of future perspectives often occurred with the intention to achieve a self-defined individual goal in the care for the child, by either the HCP or the parent. The extent of sharing future perspectives was influenced by the parents’ ability to cope with ongoing and, in particular, anticipated loss and the HCPs’ perception thereof. In addition to a goal-directed approach, a more open approach exploring mutual perspectives on future care and treatment could improve timely anticipation of future care needs of the child and family and allow parents to anticipate the future more gradually.

## Electronic supplementary material

ESM 1 (DOCX 15.3 kb)

## Abbreviations

AC: Anticipated care
ACP: Advance care planning
GDC: Goal-directed conversation
GOTJ: Guidance on the job
HCP: Healthcare professional
PPC: Pediatric palliative care
PPCT: Pediatric palliative care team
QoL: Quality of life
