# Supplementary material for: Anticipating the future of the child and family in pediatric palliative care: a qualitative study into the perspectives of parents and healthcare professionals
Source: Eur J Pediatr. 2020 Oct 8;180(3):949–57. doi: 10.1007/s00431-020-03824-z (PMC7886816; doi:10.1007/s00431-020-03824-z)
Supplement: Supplementary file 1 — (DOCX 15.3 kb) [file 431_2020_3824_MOESM1_ESM.docx]

**Topic list 1:** Anticipating future care and treatment (parents)

To what extent do parents experience anticipating care and advance care planning and to what extent do they feel a need for it?

Anticipating care

A component of palliative care is to anticipate in order to be able to (continue to) provide high quality care.

- To what extent do you experience that professionals (doctors, nurses, case managers) look ahead with you/prepare you for future situations?
- How do you experience this looking ahead? Are you able to follow this approach?
- What (goals) do the doctors have in mind for your child? What are the doctors envisioning for your child?

To what extent do you occupy yourself with the thoughts about the moment your child will decline?

With respect to your child’s situation: to what extent are you able to look ahead, and is it something that you want to/feel a need for?

- What are the thoughts you are having?
- What are your preferences? What is important to you?
- Are there things you are afraid of or things (issues/aspects) you have concerns about?
- What support do you think you will possibly need by that time?

Have there been other situations (so far) which made you look ahead or that compelled you to look ahead?

Healthcare professionals sometimes struggle to look ahead or anticipate with parents or to prepare them. Do you have any ideas or tips how professionals could best handle this?

**Topic list 2:** Anticipating future care and treatment (healthcare professional)

Anticipating care

The idea is that palliative care can benefit from anticipating care.

- Do you think anticipating care is important?
- To what extent can/could anticipating care be achieved for this child/family?
- What does anticipation/advance care mean/entail with respect to this family?
- How was anticipation/advance care formalized in this situation?
- What is/was your role in the approach to anticipating care?
- How do you view your role?
- How do you approach your role?

What do you and the child’s parents envision with respect to future care (the future)? What do you aim for?

- - Are you and the child’s parents on the same page?
  - To what extent are parents able to follow you in this approach?
  - How do you try to achieve this? What is your approach?

One of the components of anticipating care is setting up a care plan/palliative protocol.

- - To what extent is this applicable to this family?
  - How is the care plan achieved?
  - What was your role? What was the parents’ role?

Are there issues/aspects you are already considering/anticipating, but which parents have not taken into consideration yet?

- - What kind of issues/aspects are you already thinking about?
  - What is the reason you are not (yet) discussing these issues/aspects with the parents?
